# Benefits of zebra stripes: Behaviour of tabanid flies around zebras and horses

**DOI:** 10.1371/journal.pone.0210831

**Published:** 2019-02-20

**Authors:** Tim Caro, Yvette Argueta, Emmanuelle Sophie Briolat, Joren Bruggink, Maurice Kasprowsky, Jai Lake, Matthew J. Mitchell, Sarah Richardson, Martin How

**Affiliations:** 1 Department of Wildlife, Fish and Conservation Biology, University of California Davis, Davis, California, United States of America; 2 Centre for Ecology and Conservation Biosciences, College of Life and Environmental Sciences, University of Exeter, Penryn Campus, Penryn, Cornwall, United Kingdom; 3 Aeres University of Applied Sciences, Almere, Netherlands; 4 School of Biological Sciences, University of Bristol, Bristol, United Kingdom; University of Tasmania, AUSTRALIA

## Abstract

Averting attack by biting flies is increasingly regarded as the evolutionary driver of zebra stripes, although the precise mechanism by which stripes ameliorate attack by ectoparasites is unknown. We examined the behaviour of tabanids (horse flies) in the vicinity of captive plains zebras and uniformly coloured domestic horses living on a horse farm in Britain. Observations showed that fewer tabanids landed on zebras than on horses per unit time, although rates of tabanid circling around or briefly touching zebra and horse pelage did not differ. In an experiment in which horses sequentially wore cloth coats of different colours, those wearing a striped pattern suffered far lower rates of tabanid touching and landing on coats than the same horses wearing black or white, yet there were no differences in attack rates to their naked heads. In separate, detailed video analyses, tabanids approached zebras faster and failed to decelerate before contacting zebras, and proportionately more tabanids simply touched rather than landed on zebra pelage in comparison to horses. Taken together, these findings indicate that, up close, striped surfaces prevented flies from making a controlled landing but did not influence tabanid behaviour at a distance. To counteract flies, zebras swished their tails and ran away from fly nuisance whereas horses showed higher rates of skin twitching. As a consequence of zebras’ striping, very few tabanids successfully landed on zebras and, as a result of zebras’ changeable behaviour, few stayed a long time, or probed for blood.

## Introduction

The function of zebra stripes has been a source of scientific interest for over 150 years generating many hypotheses including camouflage, confusion of predators, signaling to conspecifics, thermoregulation and avoidance of biting flies [1] but contemporary data show that only one stands up to careful scrutiny [2–4]. Briefly, regarding camouflage, zebra stripes are difficult for lion *Panthera leo* and spotted hyaena *Crocuta crocuta* predators to resolve at any great distance making crypsis against mammalian predators an unlikely benefit [5]. Regarding confusion of predators, zebras do not have the sort of striping pattern that aids in confusion [6] and African lions take zebra prey disproportionately more than expected suggesting an absence of confusion effect [7]. Regarding social benefits, rates of grooming and patterns of association are no greater in striped equids than in unstriped equids [3]. Finally, there are no thermoregulatory benefits to striping based on controlled experiments using water drums [4], infrared photography of free-living herbivores [3] and logical argument in regards to flank striping [8].

Instead, there is an emerging consensus among biologists that the primary function of contrasting black and white stripes on the three species of zebras is to thwart attack from tabanids, and possibly glossinids, stomoxys and other biting muscoids based on laboratory and field experiments with striped materials [3, 9–12] and on comparative evidence [13]. In Africa where zebras live, tabanids carry diseases fatal to zebras including trypanosomiasis, equine infectious anemia, African horse sickness and equine influenza [14] and zebras are particularly susceptible to infection because their thin pelage allows biting flies to probe successfully with their mouthparts [13]. The exact mechanism by which stripes prevent flies from obtaining a blood meal is less well understood, however. Flies may fail to detect a zebra from a distance, or from close up, either as a result of misinterpreting optic flow as they approach [15], by interfering with cues that promote a landing response [9, 16], or even by disrupting the polarization signature of their host [12]. Unfortunately, detailed observations of biting flies in the vicinity of live zebras have so far been unavailable but such information would help elucidate the stage at which stripes exert an effect on host seeking by biting flies.

In this study we compare several measures of behaviour of wild tabanid horse flies around captive zebras and domestic horses living in the same habitat using direct observations and video footage. We also compare the behaviour of tabanids around horses wearing differently coloured cloth coats, report on the duration of time that tabanids spend on equids with different coloured pelage, and compare the behaviour of horses and zebras in response to biting fly annoyance.

## Methods

We studied captive plains zebras (*Equus burchelli* or *E*. *quagga*) (three females) and uniformly coloured white, grey, brown and black domestic horses (*E*. *caballus*) (three females, six males) at the Hill Livery, Dundry, North Somerset, UK (51.4100° N, 2.6469° W) in July 2016, and June and July 2017. We video recorded individual equids or took records by hand (from a distance of 1-2m) of tabanid (*Haematopota pluvialis* and *Tabanus bromius*) flies in the vicinity of their hosts; female European tabanids require a blood meal to produce eggs [17]. We also recorded equids’ reactions to these diptera. Horses and zebras were always kept in separate but adjacent fields and were both observed and videoed on the same days. The size of the zebra field was larger than two of the horse fields, similar in size to another, whereas two other horse fields were larger still; all were surrounded by hedges of similar structure and width. Pasture was the same in all fields and hay was put out intermittently in all of them.

### Study 1: Direct observations

During a total of 16.3 hours of observation of three zebras and nine horses ($\bar{\text{X}}\;\text{observation}\;\text{period}\;=\;5.\text{0min}$, range 0.7–22.3min) an observer (TC), standing on one side of the equid at 1-2m distance, recorded the number of tabanids circling around, touching, and landing on each host. Touching occurred rapidly and consisted either of the fly’s dorsum or head bumping into the pelage (<1sec), failed attempts at landing, or extremely brief landings (<2sec) followed by take-off. Landings consisted of much more prolonged contact with a fly alighting on the host for >2sec but usually for between 20 sec to 10 min. For zebras, we recorded whether tabanids touched or landed on either black or white stripes. Then, focusing attention on those tabanids that actually landed properly, we recorded the number of times that a fly walked over the pelage, or probed for a source of blood. Probing was defined as lowering the head and thorax thereby tilting the abdomen up vertically or obliquely.

### Study 2: Coats

We placed three cloth coats in turn on seven (6 males, 1 female) horses each for 30 consecutive minutes in a fully randomized order. One was a dark black Rambo Optimo stable sheet, one was a bright white Shires Equestrian Products One Performance flysheet and neck set, and the third was a black and white irregularly striped BUCAS Buzz-off zebra full neck coat. Coats were purchased commercially with no attempt at standardization of material. The outer surface of the black coat was made of “1000D ripstop polyester”; the white of “cool mesh”; and the striped of “fine mesh fabric”. We assumed that coat thickness would have no effect on its attractiveness to tabanids from a distance since the only documented structural effects of tabanid traps pertain to overall trap design [18]. Coats differed in UV reflectance with the white coat and white stripes of the “zebra” coat reflecting respectively a medium amount, and a lot of UV; the black stripes and black coat reflected very little (Figure A in S1 File). Each coat covered the horse’s rump, back, flank, belly, withers and neck but not the head or ears which were left naked. Two observers (JB and JL who knew of the hypotheses being tested) always stood 1-2m away simultaneously recording the number of tabanid flies landing on each side of the cloth coat and on the uncovered head. Animals were only watched in the open and never in the shade. Since flies were not recognized individually, the same tabanid could have landed or touched the horse multiple times but there was no a priori reason to think that this occurred differentially according to coat colour.

### Study 3: Videos of flies on equids

Video recordings were collected (JB and JL) of three zebras and seven horses (two females, five males) using a Panasonic HC-X900 camera (Osaka, Japan) always from a fixed distance of approximately 3m. From these, the length of time that tabanids remained on equid pelage during observations of one side of the animal (18.97 hours’ observations of 10 equids), their whereabouts on ten regions of the pelage (Figure B in S1 File), and whether the individual tabanid left voluntarily or was forced off by the host, was recorded from the videos (by EB and MJM who had no prior knowledge of the hypotheses).

**Fig 1:**
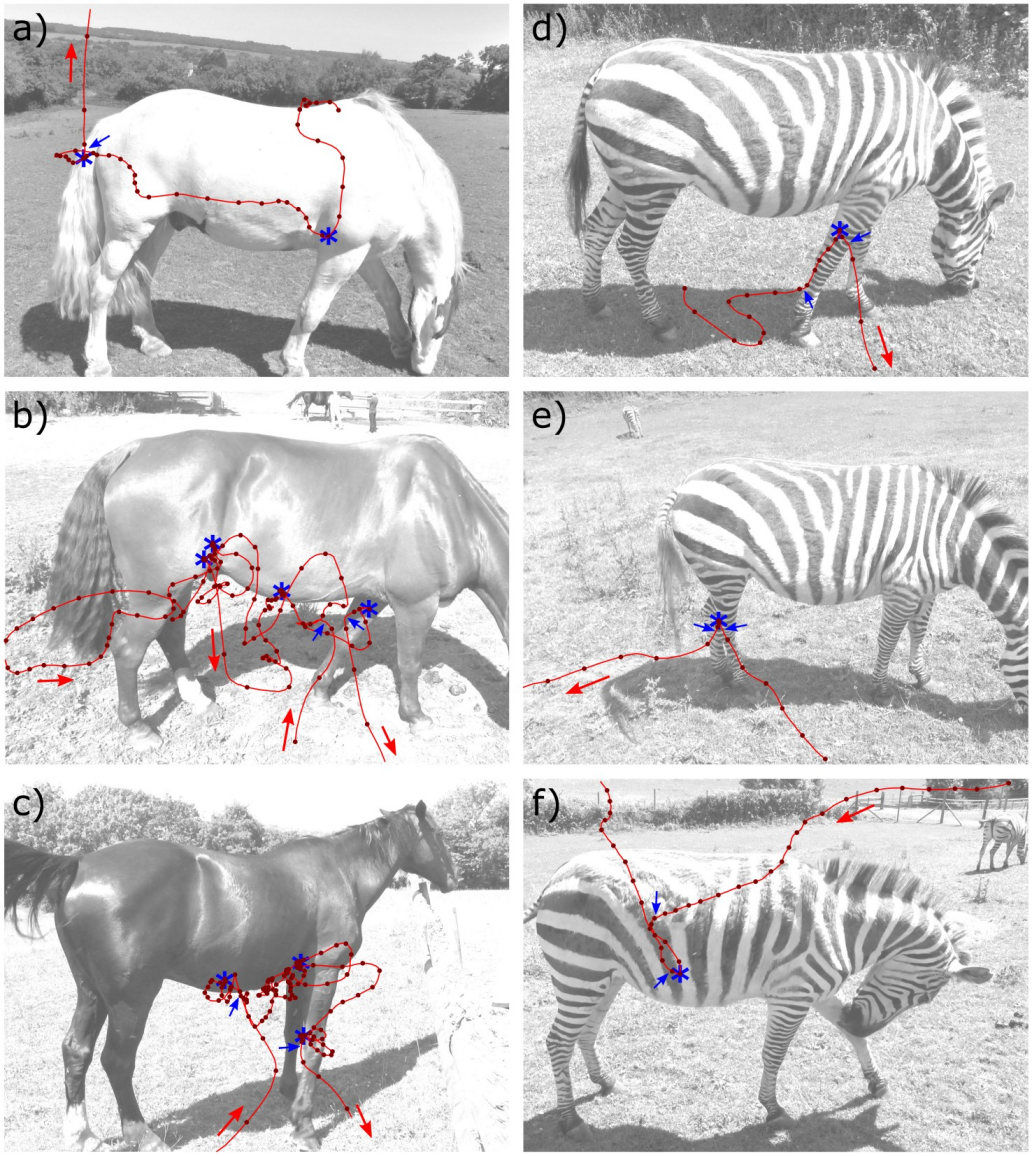
Examples of horsefly flight trajectories around domestic horses (a-c) and captive plains zebra (d-f). Red line indicates the flight path and dark red dots show position at 0.1s intervals. Red arrows indicate direction of flight. Blue stars show points of contact or landings on the equid. Blue arrows show the end position of the approach and start position of the leave phases of flight. These markers are associated with manoeuvres that show changes in both direction and speed, and where this could not be reliably identified (e.g. approach in a) the data were omitted from analysis.

Flight trajectories of tabanids in the vicinity of horses and zebras were extracted from the video recordings (MH) by marking their position every 0.02s using Matlab 2017b (Mathworks, Natick, USA). The use of custom-written zoom tools ensured that fly location could be digitized to the nearest pixel, resulting in accurate and repeatable tracks. Trajectories were divided into three segments as follows. An “approach” was the flight trajectory starting from when the fly entered the camera’s view until it performed its first deceleration and turning manoeuvre close to the pelage of the animal. This was usually a direct flight starting from the side of the camera’s field of view, but sometimes contained loops of flight at a distance from the animal (Fig 1). A “leave” was when the fly made a direct and accelerating flight away from the host ending out of the camera’s field of view. This started from the moment the fly took off from the animal, or manoeuvred away from close to the animal (Fig 1). An “investigation” was the period in between, when the fly was hovering around the pelage of the animal. (It therefore corresponds most closely to circling as recorded during direct observations). Speed, duration and tortuosity (distance traveled divided by the distance between the start and finish points) were calculated for the three trajectory segments. Note that these measures only approximate the true speed and tortuosity of the fly, as flight paths were recorded in a two-dimensional plane using a single camera and would not necessarily end in host contact. While flight speed and trajectory may have been influenced by angle of approach relative to the position of the camera, flight durations were not. In addition, flight speeds in the 0.5 seconds prior to landing and touching the hosts were determined, from which a comparison between horses and zebras was made using a linear mixed model (‘lmer’, R3.4.4, CRAN) with individual fly as a fixed effect [speed ~ time+species+(1|fly)]. P values were calculated using Satterthwaite’s method in the R package ‘lmerTest’.

### Study 4: Videos of equid behaviour

From video recordings of three female zebras and eight (3 female, 5 male) individual horses in 2017 (17.9hours total), MK and SR (who had no knowledge of the hypotheses) recorded 19 different patterns of behaviour shown by equids in response to biting flies (Table A in S1 File). from which rates per minute were calculated ($\bar{\text{X}}=\;97.3\text{min}/\text{equid}$, range 5.0–127.7min). In 2016 from videos, YA recorded parasite avoidance behaviours of the same three zebras, two different horses (1 male, 1 female) and one female plains zebra-somali wild ass (*E*. *africanus*) hybrid (4.0hours total, $\bar{\text{X}}=\;39.8\text{min}/\text{equid}$, range 15.6–61.7min).

The focus of our study was on tabanid flies of which there were many at the Livery. Since fly activity around equids could have been influenced by weather conditions (e.g. [3]), we used data from Filton, the nearest meteorological office, 17km northeast of Dundry, to approximate mean temperatures, solar radiation, maximum wind gust speeds, average windspeeds, relative humidity and cloud cover each hour that the equids were observed. These six meteorological variables are often closely associated so for each behavioural measure in our observations, we determined which of these variables was most strongly correlated with a given fly or horse behaviour using Spearman correlation coefficients. In SPSS24 we then entered that variable as a covariate in linear mixed models with individual equid as the subject for all analyses (as indicated in the text) except in Study 3 where coats were sequentially placed on the same horse; pelage was entered as a fixed factor. Analyses revealed that environmental variables showed fewer significant effects on the behaviour of tabanids than we expected. This may have been because we did not go out to the farm when the weather was wet or windy, so all our data are from calm weather conditions.

The study received approval from the Animal Welfare and Ethical Review Board at the University of Bristol (reference UB/18/074).

## Results

### Behaviour of tabanids in the vicinity of equids

Study 1 revealed no significant difference in the rates at which tabanids circled zebras or horses ($\bar{\text{X}}\text{s}=\;1.11/\text{min},\;2.25/\text{min}$ respectively, F_1, 9.291_ = 0.879, p = 0.372 controlling for individual equid and wind speed; Table Ba in S1 File). Similarly, there was no significant difference in the rate at which tabanids touched (< 2 sec) zebras and horses ($\bar{\text{X}}\text{s}=\;0.41/\text{min},\;0.72/\text{min}$, F_1, 9.020_ = 0.009, p = 0.925 controlling for individual equid and wind speed; Table Bb in S1 File). In contrast, significantly fewer tabanids landed on zebras than on horses ($\bar{\text{X}}\text{s}=\;0.26/\text{min},\;1.08/\text{min}$, F_1, 9.321_ = 5.600, p = 0.041 controlling for individual equid and wind speed; Ns = 3_zebras_, 9_horses_ in all cases; Table Bc in S1 File). Of the very few tabanids that touched or landed on zebras, 54% were on white stripes; relative areas of black and white stripes for each zebra were not calculated but were likely to be similar.

### Coat experiment

**Fig 2:**
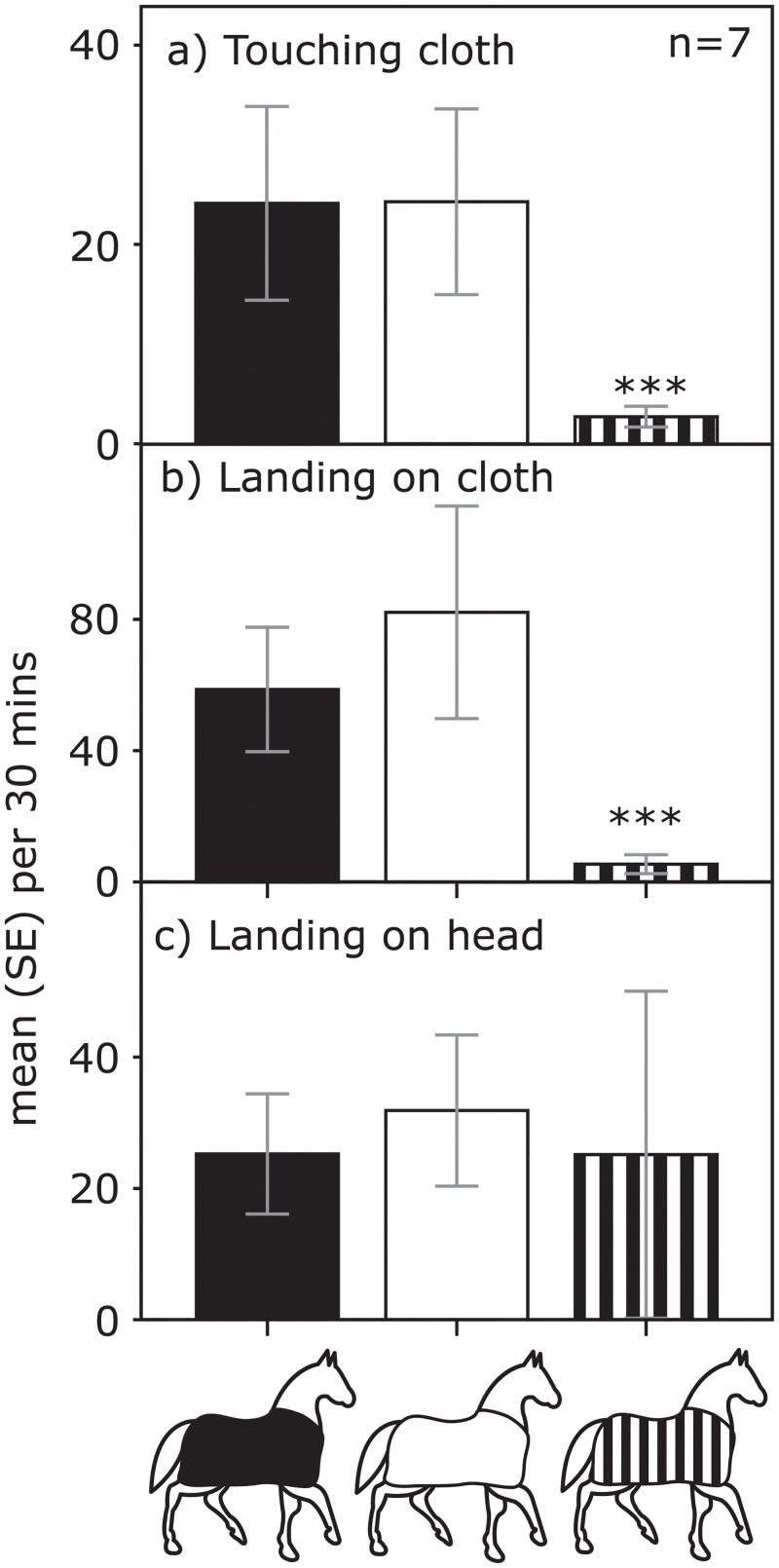
Mean (and SE) number of tabanid flies (a) touching or (b) landing on cloth coats of different shade and pattern, and (c) landing on the bare head of 7 different horses. *** = p<0.0001.

To exclude any possible influence of host odour or differential movement in attracting tabanids, we compared the number of tabanids touching and landing on both sides of different coloured cloth coats placed sequentially in random order on seven horses (study 2). Rates of touching and landing on cloth coats differed significantly (touching: N = 7, F_2, 17_ = 14.846, p < 0.0001; landing: N = 7, F_2, 2.956_ = 399.174, p < 0.0001; both controlling for temperature) with far fewer touching and landing on striped than uniform coats (Fig 2). There were no significant differences, however, in the rate at which flies landed on these horses’ naked heads (N = 7, F_2, 4.836_ = 1.021, p = 0.427 controlling for temperature, Fig 2). In summary, the zebra cloth coat had beneficial effects for the horse but the naked head suffered the same frequency of landings by tabanids.

### Flight trajectories

Compared to horses, flies approached zebras more quickly in terms of speed (median 13.21 [interquartile range 5.04] pix/frame vs. 17.76 [26.9] pix/frame respectively; Mann-Whitney U test, W = 289; p = 0.017) but their tortuosities were similar (1.19 [0.57] vs. 1.07 [0.48] respectively; W = 540; p = 0.18). In contrast, tabanids spent a greater amount of time investigating horses than zebras (1.42 [2.18] s vs. 0.76 [1.88] s respectively; W = 586; p = 0.036; Fig 1), although their flight speeds and tortuosities did not differ significantly (speed: 5.8 [4.4] pix/frame vs. 5.7 [4.0] pix/frame respectively; W = 486; p = 0.54; tortuosity: 2.5 [3.0] vs. 2.6 [5.0] respectively; W = 431; p = 0.85). Flies flew away significantly faster from zebras than from horses (25.1 [22.0] pix/frame vs 15.3 [7.6] pix/frame respectively; W = 252; p = 0.0030) but tortuosities did not differ significantly (vs. 1.0 [0.08] vs 1.1 [0.3] respectively; W = 330; p = 0.37; N_flies_ = 22–37 throughout).

**Fig 3:**
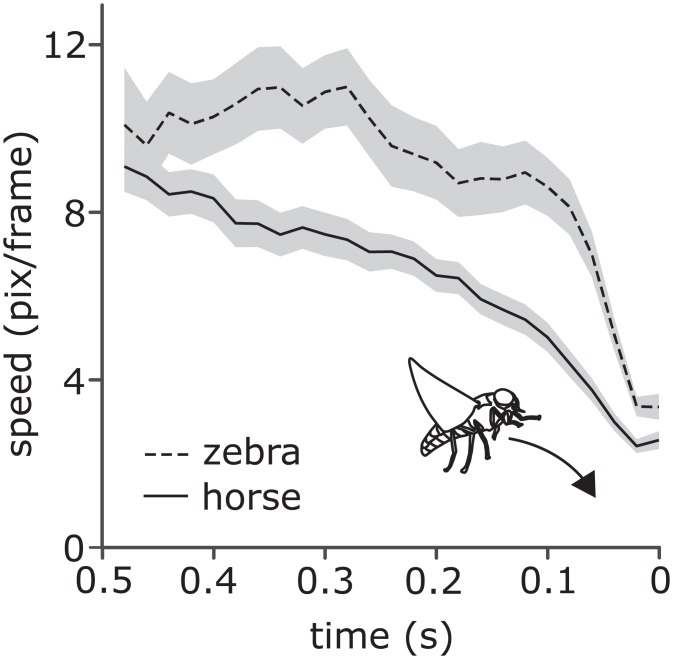
Mean flight speed (in pixels per video frame) of tabanids during the final 0.5 seconds of approaching horses (solid line) and zebras (dashed line). Grey area = ± SE. N_horse approaches_ = 39; N_zebra approaches_ = 26.

Focusing to the 0.5 s period prior to actually contacting equids’ coats, we noticed that tabanids approaching zebras failed to decelerate in a controlled fashion towards the end of their flight trajectories whereas they steadily decelerated before landing or touching horse pelage (t = 3.30; df = 61.3; p = 0.0016 taking individual fly into account; Fig 3). Moreover, flies often simply bumped into zebras but fail to land or fly away: data from study 1 showed that a significantly greater proportion of tabanids touched zebras as compared to horses ($\bar{\text{X}}\text{s}=\;21.7\%,\;14.7\%\;$ respectively, F_1, 8.027_ = 5.659, p = 0.045 controlling for individual equid and solar radiation) whereas, conversely, a significantly lower proportion landed on zebras than on horses ($\bar{\text{X}}\text{s}=\;9.9\%,\;27.9\%$ respectively, F_1, 7.813_ = 47.172, p < 0.0001 controlling for individual equid and maximum wind gusts), almost one third fewer on average.

### Behaviour of tabanids once on the host

If a tabanid had actually landed on an equid, study 1 showed that there was no significant difference in the number of instances in which they walked across zebra pelage than across horse pelage ($\bar{\text{X}}\text{s}\;=\;0.07\;\text{moves}$/landing vs 0.33 moves/landing respectively, F_1, 11.158_ = 0.895, p = 0.364 controlling for individual equid and solar radiation) but significantly fewer probed the skin of zebras than of horses ($\bar{\text{X}}\text{s}\;=\;0\;\text{probes}$/landing vs 0.93 probes/landing respectively, F_1, 10.306_ = 5.481, p = 0.041 controlling for individual equid and maximum wind gust). In fact, we saw no tabanid probe zebra skin during 5.3 hours total of direct zebra observation as opposed to 239 instances during 11.0 hours of observing horses.

**Fig 4:**
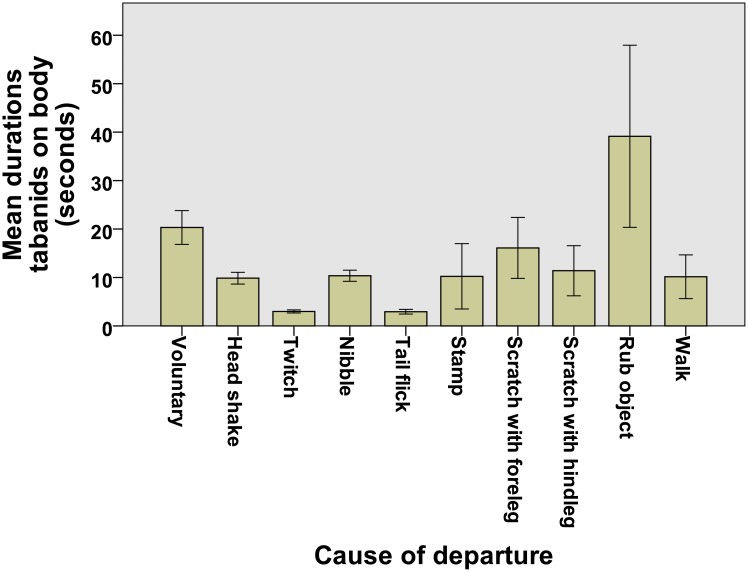
Mean (and SE) durations that tabanids spent on equids’ bodies separated by method of forcible eviction. On the far left are durations when tabanids left voluntarily.

Study 3 showed that tabanids spent longer periods of time on the face, neck and to a lesser extent forelegs of their equid hosts (here we had to combine both equid species as so few tabanids landed on zebras, Figure C in S1 File). These regions of the body are impossible for an equid to access with its mouth or tail but they may also be areas with shallow pelage [19]. Equids forcibly removed tabanids using a variety of methods (study 4), the most successful of which (in terms of reducing time spent on the host) were skin twitching and tail swishing (Fig 4). Interestingly, every behaviour, except rubbing against objects, reduced the amount of time that tabanids spent on the host compared to the fly leaving voluntarily (see Fig 4).

From study 3 we found that the average length of time that tabanids spent on zebras was far less than on horses (Table 1). On average, tabanids spent 1.20 sec (SE = 0.91, N = 5 instances) on zebras but an average of 10.06 sec (SE = 0.08, N = 886 instances) on horses (Mann-Whitney U test, p = 0.012).

**Table 1:** Average time that tabanids spent on each equid taken from video recordings. Also shown are mean landings per minute and hours being filmed.

	Zebra	Zebra	Zebra	White horse	Grey horse	Brown horse	Brown horse	Brown horse	Black horse	Black horse
Name	Spot	Nick	Shadow	Snowy	Bertha	Lizzie	Ginger	Phoenix	Tom	Posh
Sex	F	F	F	M	F	F	M	M	M	M
X time on host in secs	1.33	0	1.00	4.94	25.75	39.26	10.63	41.12	35.02	23.52
X landings / minute	0.02	0	0.18	1.03	1.17	1.27	0.76	1.38	2.88	2.47
Time filmed in hours	1.99	1.79	1.86	1.77	1.09	2.12	2.25	1.90	2.14	2.06

### Response of equids to biting flies

Study 4 showed that striped and unstriped equids responded to fly annoyance using very similar behaviour patterns (Table 2) All equids were attended by tabanids to varying extents, but given that far more tabanids landed on horses than on zebras (see above) it was perhaps unsurprising that rates of virtually all behaviours used to rebuff flies were greater for horses, and in those exceptions, mean differences were very small. Specifically, most sorts of twitching were greater in horses than in zebras (shoulder twitching $\bar{\text{X}}\text{s}\;=\;13.49/\text{min},\;3.13/\text{min}$ respectively, F_1, 9.141_ = 13.173, p = 0.005 controlling for individual equid and windspeed; withers $\bar{\text{X}}\text{s}\;=\;3.98/\text{min},\;0.13/\text{min}$ respectively, F_1, 9.548_ = 19.948, p = 0.001 controlling for individual equid and windspeed, belly $\bar{\text{X}}\text{s}\;=\;5.35/\text{min},\;0.96/\text{min}$ respectively, F_1, 8.694_ = 11.300, p = 0.009 controlling for individual equid and maximum wind gusts; total skin twitching $\bar{\text{X}}\text{s}\;=\;24.28/\text{min},\;5.02/\text{min}$ respectively, F_1, 9.435_ = 26.474, p = 0.001 controlling for individual equid and windspeed). That rates of stamping, nibbling and scratching were greater for horses but not significantly so may reflect the fact that the latter two sets of behaviours often followed fly departures rather than being instigated by them.

**Table 2:** Average number of times per minute that individual equids performed behaviours in response to biting flies.

Behaviour	Zebras	Horses	F	df	P-value
Head shake*	2.09	4.60	4.697	1, 9.403	0.057
Ear twitch+	0.77	1.38	1.332	1, 8.484	0.280
Shoulder twitch*	3.13	13.49	13.173	1, 9.141	**0.005**
Withers twitch*	0.13	3.98	19.948	1, 9.548	**0.001**
Belly twitch#	0.96	5.35	11.300	1, 8.694	**0.009**
Leg twitch+	0.03	0.07	0.609	1, 7.015	0.461
Total twitch*	5.02	24.28	26.474	1, 9.435	**0.001**
Foreleg stamp^	0.21	0.28	0.425	1,7.305	0.534
Hindleg stamp^	0.27	0.39	0.727	1, 8.158	0.418
Total stamp!	0.49	0.68	0.551	1, 7.408	0.481
Nibble shoulder*	0.21	0.98	4.176	1, 9.448	0.070
Nibble belly*	0.11	0.38	1.407	1, 10.219	0.262
Nibble leg	0.07	0.05	0.611	1, 7.464	0.459
Nibble rump	0.05	0.04	0.128	1, 8.846	0.728
Total nibble*	0.43	1.45	3.097	1, 9.531	0.110
Kick!	0	0.01	0.089	1, 8.177	0.772
Foreleg scratch!	0.29	0.51	0.729	1, 8.558	0.416
Hindleg scratch@	0.20	0.26	0.015	1, 8.395	0.904
Object scratch!	0	0.04	0.822	1, 10.397	0.385
Tail flick!	111.72	43.20	48.461	1, 9.597	**<0.0001**
Snap@	0.42	0	3.056	1, 8.736	0.115
Escape level	0.2	0	χ2 = 44.942	2	**<0.0001**

Also shown are F, df and P-values controlling for individual equid and

* windspeed,

^+^ solar radiation,

^ humidity,

^!^ temperature,

^#^ maximum wind gust,

^@^ cloud cover.

Escape behaviour is tested with a Chi-square test, Significant results shown in bold.

In contrast, zebras tail-flicked more frequently than horses ($\bar{\text{X}}\text{s}\;=\;111.72,\;43.20/\text{min}$ respectively, F_1, 9.597_ = 48.461, p < 0.0001 controlling for individual equid and temperature), and in response to flies, they were less likely to remain stationary (Ns = 147, 285 video clips respectively, 85.0% vs 100% respectively) and instead walked away briskly (10.2% vs 0%) or ran away from flies (4.8% vs 0%) (Chi square = 44.94, df = 1, p < 0.0001, combining these move away categories).

## Discussion

That stripes act to deter landings of biting flies has been suspected for over 75 years [20] and subsequently confirmed by repeated field and laboratory experiments [3, 9–12] although the effect has never been examined closely in live striped equids. We observed the behaviour of tabanids around live zebras living on a horse farm in Somerset, UK. European horseflies may differ in behaviour from congeners in Africa where zebras live but nevertheless there are a great many species of horse flies in Africa [21] with many being attracted to zebra pelage [3]. Insect visual systems are highly conserved across taxa [22] and there are no independent reasons to think that the visual system of European tabanids will be substantially different from those in Africa.

### Approaching the host

Regarding the behaviour of tabanids close to their hosts, we noted that striped equids did not experience reduced rates of European tabanids circling around them. This suggests that stripes do not thwart attraction of tabanids from even a little distance away. Gibson [10] investigated the attractiveness of striped targets for glossinids in the field. She caught more tsetse flies on transparent side panels than on her adjacent horizontally striped target leading her to a similar conclusion: her tseste flies actively avoided horizontal stripes at the conclusion of their flights (but see [9]).

An important finding made here supported this idea that stripes do not thwart approach from a distance. There was no significant difference in rates of landings on horses’ naked heads even though zebra cloth coats received fewer landings per unit time than black or white cloth coats. This suggests that stripes had little effect at a distance but, once close up, stripes prevented landings, with flies turning their attention to the naked head instead. This was not because they were unable to penetrate the cloth coats: many flies landed on the black and white coats and spent time there. Differences in UV reflectance (Figure A in S1 File) could not explain these findings either. White coats and white stripes reflected UV but not black coats, black stripes or straps affixing the white coats. If UV attracted flies, we would expect many flies on the white coat, intermediate numbers on the striped coat, and few on the black coat. If UV repelled flies, we would expect the reverse. We found neither of these patterns in our data.

Other studies have found that compared to black surfaces, white surfaces are less attractive to tabanids [23–25] and we suspect that the white coats’ blue holding straps (3 around the belly, 3 around the neck, 2 around the chest) were attractive to tabanids. On the four horses in which we had data excluding landings on straps from landings on white coats, an average of 20.3 flies landed per half hour on the white coats but 41.3 on the black coats.

In retrospect, the failure to find differences in approach towards striped and unstriped objects is unsurprising because tabanids are thought to use odour rather than visual cues to locate hosts at a distance [26] and only switch to vision when close up. Indeed past calculations suggest that flies are only likely to discern zebra stripes at a distance of <20m [16], before that it will simply look grey to them. Waage [9] estimated that tsetse flies resolve stripes at 4.4m distance away, for instance. Our data suggest that tabanids may only be able to resolve stripes at a 2m distance since they appear relatively unaffected further away than this (see Fig 1).

Our data show that tabanids approached zebras more rapidly than horses suggesting that they did not slow down as they approached or else were more motivated. The second idea seems unlikely because once tabanids had arrived at the host and were close to it they spent shorter periods of time investigating zebras than horses although flight speeds and tortuosities did not differ (Note durations could not be influenced by our 2-D imaging).

### Contact with the host

Rates of briefly touching striped coats were considerably lower than for either white or black cloth coats, although rates of touching did not differ between live zebras and horses. Moreover, successful landing rates were consistently lower on striped coats compared to black or white coats as well as on live zebras than live horses. Importantly, we discovered that tabanids failed to decelerate in the terminal stages of their flights before contacting zebras but not horses. In the last half second they flew faster before landing on or touching a zebra than a horse suggesting they did not see the target, or did not regard the striped surface as an appropriate place to land, or were confused somehow by the stripe pattern perhaps because it disrupted optic flow [15]. Indeed, the proportion of tabanids that simply touched live zebras was significantly greater, and the proportion landing was significantly lower than for horses. The mechanism by which controlled landings operate in tabanids is unknown but fruit flies and bees hold the angular velocity of the image on the eye constant in order to regulate flight speed [27]. Interspersed black and white stripes are likely to prevent these accurate assessments of angular velocity of looming objects in ways that demand further investigation (see [28, 29]).

### Behaviour of the host

In addition to stripes preventing landing attempts, we found that when a tabanid did land, it spent less time on a zebra than on a horse and was less likely to probe for a blood meal per landing. This was unlikely to be mediated by hair colour since flies had already landed; instead it implies that zebras were using behavioural mechanisms to stop parasites feeding on them (see Table 2). Zebras did indeed, show far higher rates of both tail flicking and running away from tabanid annoyance than did domestic horses. High rates of tail flicking have been recorded both in plains zebras living in the wild as well as high rates in captive zebra species in the Berlin zoo [3]. However this cannot be the only reason that flies stayed such a short time on zebras because zebra tails can only reach 25% of the length of the head and body [3], see also [29]. Moreover, we noticed that the three captive zebras at Dundry would stop feeding if a tabanid touched or circled them repeatedly and would snap at it with their mouths or eventually run off, whereas these behaviours were never observed in horses (Table 2).

### Conclusion

In summary, multiple lines of evidence indicate that stripes prevent effective landing by tabanids once they are in the vicinity of the host but did not prevent them approaching from a distance. In addition, zebras appear to use behavioural means to prevent tabanids spending time on them through constant tail swishing and even running away. As a consequence of both of these morphological and behavioural defenses, very few tabanids are able to probe for a zebra blood meal as evidenced by our data.

Three additional but more speculative points may be made in closing. First, we found that rates at which tabanids circled and touched a single grey horse were lower than for zebras although landing rates did not differ significantly (Table Ba-c in S1 File). This was in contrast to comparisons between zebras and horses of other colours where circling and touching rates did not differ but where zebras enjoyed fewer landings per unit time. More work on grey pelage in relation to fly annoyance is clearly needed because stripes will appear grey from a distance to flies (Text A in S1 File).

Second, we found that there was no difference in rates at which tabanids moved across the surface of striped or uniform coats. Since black and white stripes give off different heat loads during the day [30–32], they could possibly confuse a tabanid if it tried to locate a capillary by thermal sensitivity (although we have no evidence that they do this). If stripes did prevent a tabanid from locating a capillary we might expect greater rates of searching zebra pelage but this was not the case.

Third, extremely high rates of tail flicking were seen in the zebra/wild ass hybrid at Dundry (Text B in S1 File) similar to that observed in African wild asses at the Tierpark Zoo (table 5.3 in [3]) suggesting that tail flicking may in part be a species-specific trait. Striping is also a species specific trait and also under partial genetic control (as witnessed by mother-offspring striping similarities, for example, TC pers obs). Therefore both morphological and behavioural anti-parasite defense strategies appear to be under strong selection in zebras.

## Supporting information

S1 File Text A. The significance of grey pelage. Text B. 2016 data. Table A. Equid behaviour patterns used to dislodge tabanids. Table Ba. Rates of tabanids circling. Table Bb. Rates of tabanids touching. Table Bc. Rates of tabanids landing. Figure A. Percentage reflectance plotted against wavelength for the horse coats. Figure B. Areas of the body used for scoring where tabanids landed. Figure C. Mean durations (and SEs) that tabanids spent on different areas of equids’ bodies.
(DOCX)

S2 File Original data. (ZIP)
