# Supplementary material for: Benefits of zebra stripes: Behaviour of tabanid flies around zebras and horses
Source: PLoS One. 2019 Feb 20;14(2):e0210831. doi: 10.1371/journal.pone.0210831 (PMC6382098; doi:10.1371/journal.pone.0210831)

**Supplementary material: Benefits of zebra stripes: behaviour of tabanid flies around zebras and horses (Caro et al.)**

**S1 File. Text A. The significance of grey pelage. Text B. 2016 data. Table A. Equid behaviour patterns used to dislodge tabanids. Table Ba. Rates of tabanids circling. Table Bb. Rates of tabanids touching. Table Bc. Rates of tabanids landing. Fig A. Percentage reflectance plotted against wavelength for the horse coats. Fig B. Areas of the body used for scoring where tabanids landed. Fig C. Mean durations (and SEs) that tabanids spent on different areas of equids’ bodies.**

S1 File. Text A. The significance of grey pelage

At a distance of >20m black and white stripes must appear grey to a tabanid (Britten et al. 2016) so we wanted to determine whether it was the overall colour of zebras rather than the stripes themselves that might reduce tabanid annoyance. Therefore we compared tabanid responses to zebras to the only available grey horse at Hill Livery. We found that significantly fewer tabanids circled the dappled grey mare than the zebras (t = 2.763, p = 0.027; Table Ba), or touched her (t = 3.092, p = 0.011; Table Bb) but there was no significant difference in the number of tabanids landing on zebras or the dappled grey (t = 0.943, p = 0.380) whereas significantly more landed on horses of other pelage colour compared to zebras (see Table Bc). This was in contrast to comparisons between zebras and horses of other colours where circling and touching rates did not differ but where zebras enjoyed fewer landings per unit time. (It is similar to Gibson’s (1992) finding that grey and vertically striped targets caught similar numbers of *Glossina pallidipes*).

We only observed one grey horse and it was dappled grey (to the human eye) with many contrasting boundaries between small blotches of white, light grey and dark grey. If grey is difficult to discern at a distance perhaps because it blends in with the sky, this would be roughly the same as black and white striping that will initially appear grey to an approaching dipteran (see Waage 1981; Gibson 1992). Nonetheless, flies have UV-sensitive photoreceptors and the sky is a strong source of UV, whereas pelage typically has low UV-reflectance.

S1 File. Text B. 2016 data

YA collected from a smaller sample of two different horses but the same three zebra individuals in 2016. That year each horse showed elevated rates of trying to dislodge flies exclusive of tail flicking (36.31/min and 35.80/min) compared to the three zebras (14.63/min, 10.41/min and 20.78/min), but zebra tail flicking rates were generally higher (horses: 43.87/min, 48.76/min, zebras: 90.74/min, 20.98/min, 98.19/min). Interestingly, the plains zebra-somali wild ass with striped legs but fawn-grey body showed an intermediate rate of dislodging flies (26.28/min) but tail flicking rates even greater than zebras (120.05/min).

| **Head shake** | Twisting/turning head in horizontal direction two or more times. |
| --- | --- |
| **Ear twitch** | Rapid movement of an ear to the sides, to the front and back, or in a circular motion as a single or multiple movement recorded as one twitch. |
| **Shoulder twitch** | Rapid movement of shoulder muscles (between top of leg to withers). |
| **Withers twitch** | Rapid movement of the withers muscles (behind the neck and over shoulder blade). Only recorded when twitch started at withers. |
| **Belly twitch** | Rapid movement of one of the belly muscles between shoulder and hip. |
| **Leg twitch** | Rapid movement of the leg muscle. Only recorded when only the leg muscle itself twitched, because otherwise muscles just moved along with a shoulder twitch. |
| **Total twitch** | Total number of shoulder, belly, withers, leg and ear twitches. |
| **Foreleg stamp** | Foreleg hoof brought heavily down on the ground - harder/faster than a normal step. |
| **Hindleg stamp** | Hindleg hoof brought heavily down on the ground - harder/faster than a normal step. |
| **Total stamp** | Total foreleg and hindleg stamps. |
| **Nibble Shoulder** | Nibble shoulder, touching the area above the legs but below the withers with the mouth/teeth. |
| **Nibble belly** | Nibble belly, touching the area between the shoulder and back leg/rump with the mouth/teeth. |
| **Nibble leg** | Nibble leg, touching any legs (below the shoulder) with the mouth/teeth. |
| **Nibble rump** | Nibble rump, touching the rump with the mouth/teeth. |
| **Total nibble** | Total leg, rump, shoulder and belly nibbles. |
| **Kick** | Aggressive movement with the hindlegs, hoof doesn't come into contact with the body or ground during the kick. |
| **Foreleg scratch** | Using the hooves on the forelegs to scratch the body or using the foreleg to scratch head/neck. |
| **Hindleg scratch** | Using the hooves to scratch the body, usually the belly. |
| **Object scratch** | Using an object to scratch an area to remove a fly. |
| **Tail Flick** | Movement of tail where the end of the tail moves past the leg, or the dock of the tail moves enough for the whole tail to move. Movement to each side of the body = 1 flick. |
| **Snap** | Vigorous head shakes where the mouth is seen to 'snap' as if trying to catch the flies in their mouth (seen only in zebras). |
| **Escape** | Walks away rapidly (1), or runs away (2), in order to escape flies following one or more behaviours shown above. |

S1 File. Table A. Equid behaviour patterns used to dislodge tabanids.

S1 File. Table Ba. Rates of tabanids circling around plains zebras and horses of different colours controlling for individual equid and windspeed; statistics show comparisons to zebras.

|  | Mins observed | Number of sessions | Number of equids | *X* circle/min | Df | t | *p-value* |
| --- | --- | --- | --- | --- | --- | --- | --- |
|  |  |  |  |  |  |  |  |
| Zebras | 318.17 | 68 | 3 | 1.11 |  |  |  |
|  |  |  |  |  |  |  |  |
| White horse | 104.17 | 20 | 1 | 2.95 | 1, 3.960 | 1.842 | *0.140* |
| Grey horse | 62.03 | 12 | 1 | 0.45 | 1, 7.136 | -2.763 | *0.027* |
| Brown horses | 274.45 | 50 | 4 | 2.32 | 1, 4.801 | 2.045 | *0.099* |
| Black horses | 218.97 | 44 | 3 | 2.81 | 1, 6.719 | 1.389 | *0.209* |

S1 File. Table Bb. Rates of tabanids touching plains zebras and horses of different colours controlling for individual equid and windspeed; statistics show comparisons to zebras.

|  | Mins observed | Number of sessions | Number of equids | *X* touch/min | Df | t | *p-value* |
| --- | --- | --- | --- | --- | --- | --- | --- |
|  |  |  |  |  |  |  |  |
| Zebras | 318.17 | 68 | 3 | 0.41 |  |  |  |
|  |  |  |  |  |  |  |  |
| White horse | 104.17 | 20 | 1 | 0.94 | 1, 5.139 | 0.979 | *0.372* |
| Grey horse | 62.03 | 12 | 1 | 0.08 | 1, 10.356 | -3.092 | *0.011* |
| Brown horses | 274.45 | 50 | 4 | 0.70 | 1, 6.477 | 0.760 | *0.474* |
| Black horses | 218.97 | 44 | 3 | 0.89 | 1, 9.419 | 0.160 | *0.876* |

S1 File. Table Bc. Rates of tabanids landing on plains zebras and horses of different colours controlling for individual equid and windspeed; statistics show comparisons to zebras.

|  | Mins observed | Number of sessions | Number of equids | *X* land/min | df | t | *p-value* |
| --- | --- | --- | --- | --- | --- | --- | --- |
|  |  |  |  |  |  |  |  |
| Zebras | 318.17 | 68 | 3 | 0.26 |  |  |  |
|  |  |  |  |  |  |  |  |
| White horse | 104.17 | 20 | 1 | 1.53 | 1, 3.800 | 3.737 | *0.022* |
| Grey horse | 62.03 | 12 | 1 | 0.32 | 1, 6.440 | -0.943 | *0.380* |
| Brown horses | 274.45 | 50 | 4 | 0.99 | 1, 4.512 | 3.184 | *0.028* |
| Black horses | 218.97 | 44 | 3 | 1.29 | 1, 6.166 | 3.154 | *0.019* |

S1 File. Fig A. Percentage reflectance plotted against wavelength for the horse coats. White and black stripes were from the “zebra” coat. Strap reflectance was taken from the white coat only.


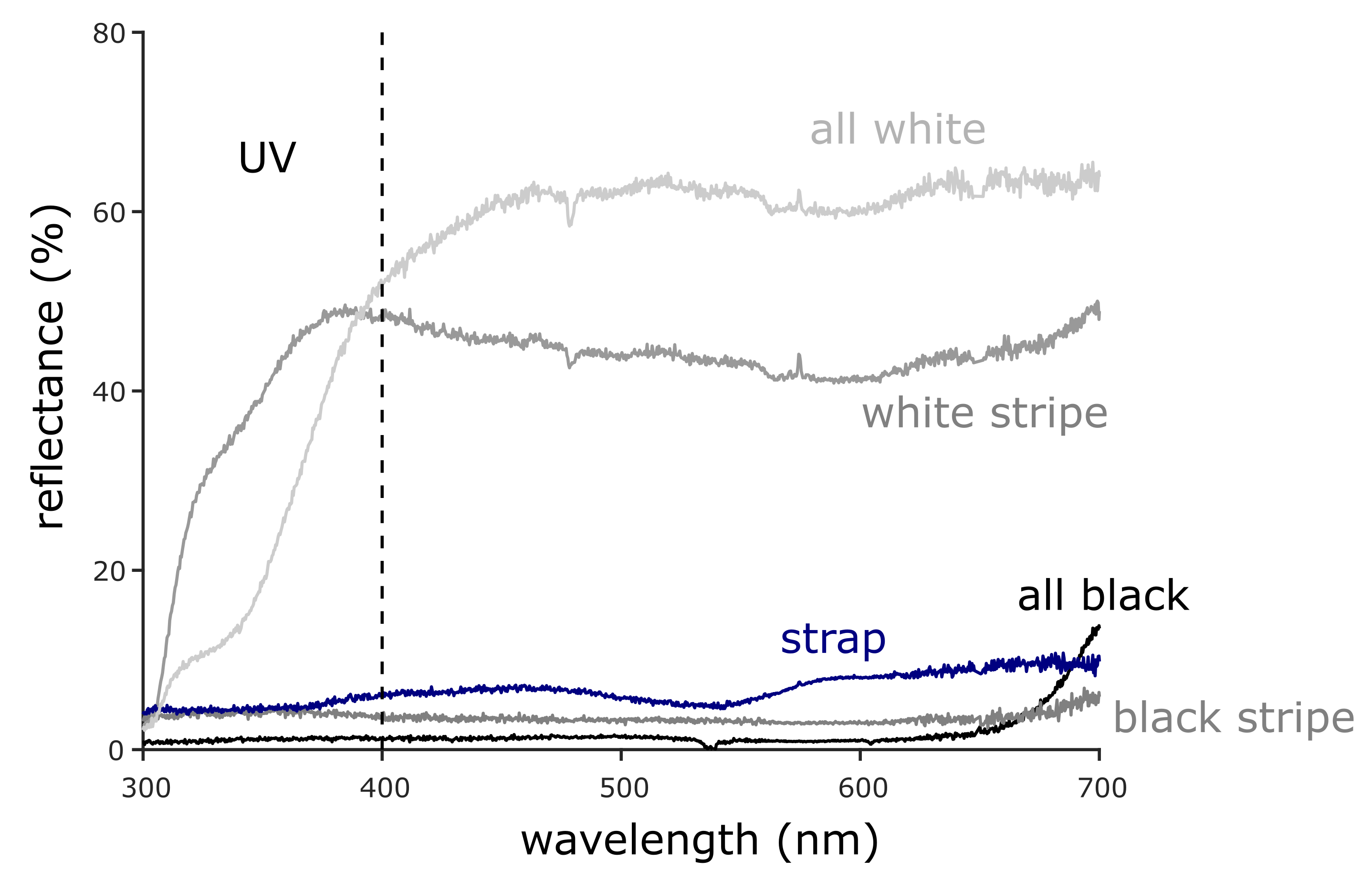


S1 File. Fig B. Areas of the body used for scoring where tabanids landed.

**
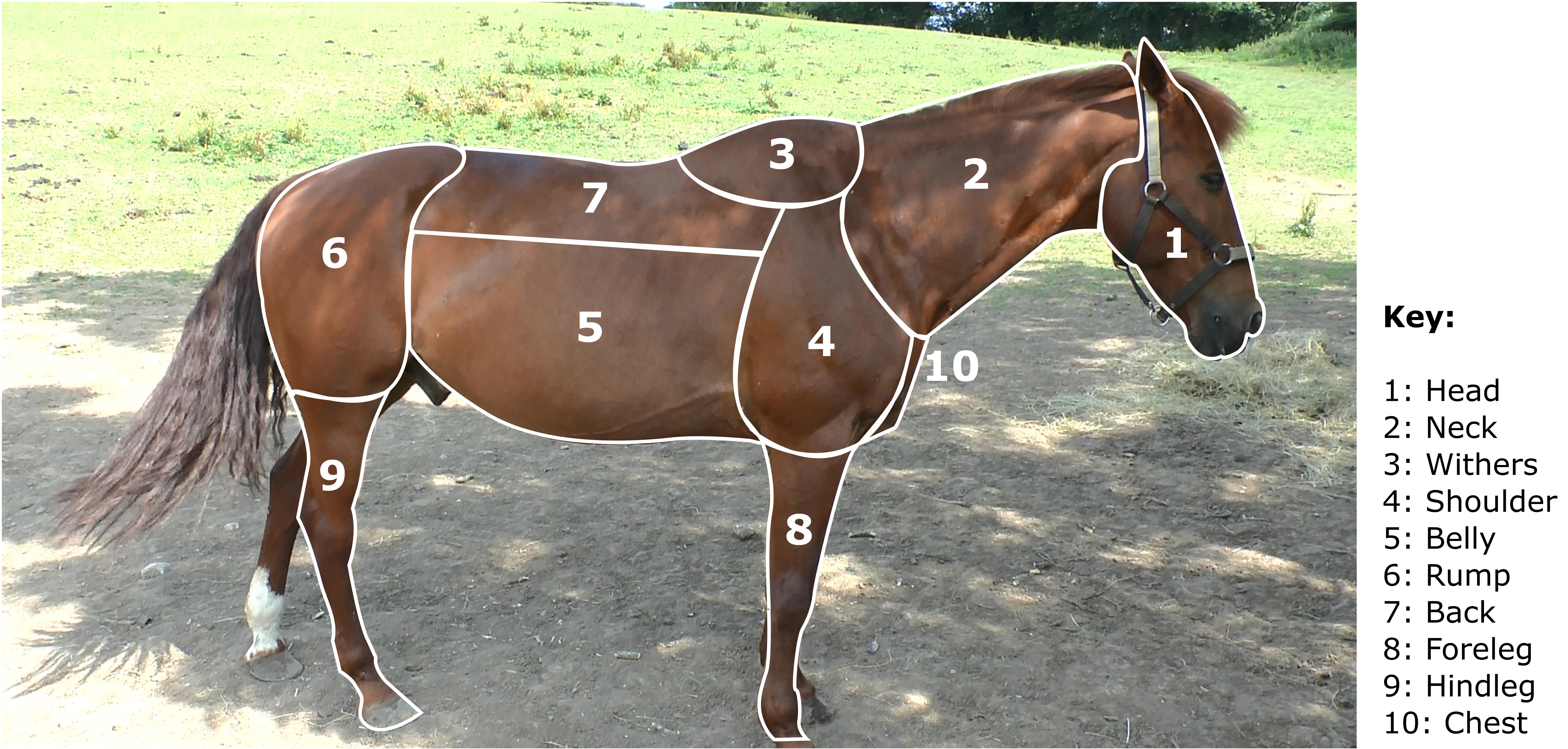
**

S1 File. Fig C. Mean durations (and SEs) that tabanids spent on different areas of equids’ bodies (extracted from videos).


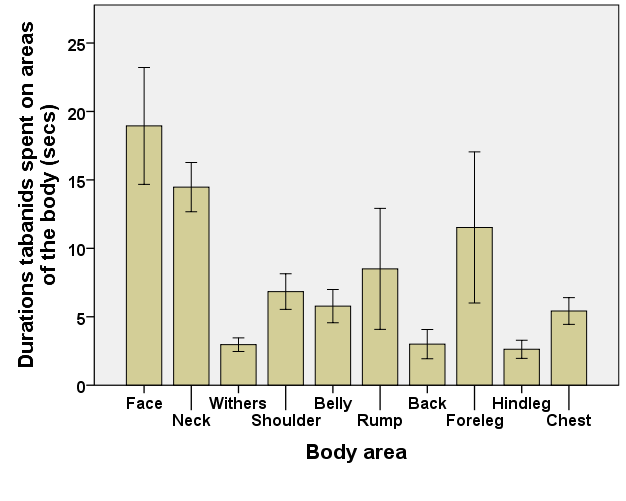

Supplement: S1 File — Text A. The significance of grey pelage. Text B. 2016 data. Table A. Equid behaviour patterns used to dislodge tabanids. Table Ba. Rates of tabanids circling. Table Bb. Rates of tabanids touching. Table Bc. Rates of tabanids landing. Figure A. Percentage reflectance plotted against wavelength for the horse coats. Figure B. Areas of the body used for scoring where tabanids landed. Figure C. Mean durations (and SEs) that tabanids spent on different areas of equids’ bodies. (DOCX) [file pone.0210831.s001.docx]
